# Supplementary material for: A Qualitative Study Supporting Optimal Nutrition in Advanced Liver Disease—Unlocking the Potential for Improvement
Source: Nutrients. 2024 Jul 24;16(15):2403. doi: 10.3390/nu16152403 (PMC11313738; doi:10.3390/nu16152403)
Supplement: Supplementary file 1 [file nutrients-16-02403-s001.zip › nutrients-3100830-supplementary.pdf]

A qualitative study supporting optimal nutrition in Advanced Liver Disease – unlocking the potential for improvement.

Table S1. Glossary of Terms

Term	Description
Nutrition	The process of taking in food and key vitamins and minerals to keep healthy
Physical health	Physical aspects of health; the normal functioning of the body
Quality of life/wellbeing	A standard of happiness, comfort and health; ability to complete activities that you like to do
Social wellbeing	Maintenance of healthy relationships with surrounding people; ability to interact and complete activities that add to quality of life
Emotional wellbeing	Emotional resilience; recognition of stress and ability to implement healthy strategies to deal with stress long term; Handle stresses and adapt to hardships; ability to produce positive emotions
Ascites	Fluid and swelling of the abdomen
NG tube	Nasogastric tube that is inserted through the nose into the stomach to allow for direct feeding
Depression	Feeling down, feeling flat, feeling depressed, low mood
Appetite	Desire to eat or for food
Jaundice	Yellowing of skin and whites of the eyes due to medical conditions (e.g. liver disease)
Dietician	Comments regarding the role of the Dietician
Nausea	Feel like you are going to be sick
Vomiting	Ejecting matter from the stomach via your mouth
Patient	Person, participant
Drowsiness	Drowsy, sleepy
Nutrition screening tool	Tool to assess risk of malnutrition
Sleep disturbance	Sleep problems, insomnia, unable to sleep
Carer burden	Impacts associated with caring for someone
Malnutrition	A condition that results from inadequate nutrition or impaired utilization of nutrients
Liver clinic	A facility that specializes in liver disease with a multidisciplinary team
Bloating	A swollen state caused by the retention of gas or fluid, commonly in the abdomen
Mediterranean diet	Traditional diet in Mediterranean countries high in vegetables, olive oil and moderate protein
Bowels	Intestines, part of the digestive system
Energy/energy levels	The strength required to perform physical or mental activity
Finances	Money required for living
Dental issues	Problems with teeth that may affect ability to eat
Dentures	A removable plate with one or more artificial teeth
Healthcare team	Doctor, Nurse, Social Worker, Dietician; Liver health care team; Supportive care team.
Liver disease	Damage to the Liver

Advanced liver disease	The term used to describe chronic liver disease that has progressed to near cirrhosis, or cirrhosis
Nutrition care	Care focused around patients receiving adequate nutrition
Standard care	Care provided in the usual inpatient/outpatient hospital setting
Cirrhosis	End Stage Liver Disease, Childs Pugh B or C score, high MELD score
Decompensated liver disease	Symptomatic complications of liver disease
Compensated liver disease	Well controlled, the liver is still able to function adequately, the condition is stable
Referral process	Process of referring patients to other health professionals/disciplines
Hospital	Names of sites
Cognition	Acquiring knowledge and understanding through thought, senses and experience
Support	Help and assistance, care
Non physiological factors	Includes food security, culture, dentition, psychological wellbeing, social/emotional wellbeing
Financial security	Having adequate funds for cost of living (housing food, electricity, health care, etc)
Food security	Having physical and economic access to sufficient, safe, and nutritious food
Fatty liver	A build-up of fats in the liver that can damage the organ
Culture/cultural background	The context of someone's background involving ethnicity, family, race, socioeconomic status, gender, etc.
Nurse	Comments regarding the role of the Nurse
Doctor	Comments regarding the role of the Doctor
Symptom	Reported physical, psychological, emotional or social problem
Supportive care	Holistic care provided by a multidisciplinary team for patients with life limiting chronic illness, and their caregivers
Researcher	A person who carries out academic or clinically-based research
Carer/caregiver	Husband, partner, sister, family, friend
Caring	To care for someone
Patient centred	Person centred, patient at the centre of their care planning
Symptom burden	Burden of Symptoms to patient, impacts on quality of life, severity of symptom burden
